# The complete chloroplast genome of *Gentaina urnula* (Gentianaceae)

**DOI:** 10.1080/23802359.2019.1622463

**Published:** 2019-07-10

**Authors:** Qiao Yang, Chunlin Chen, Li Huang, Wenjie Yang, Quanjun Hu

**Affiliations:** Key Laboratory of Bio-Resource and Eco-Environment of Ministry of Education, College of Life Sciences, Sichuan University, Chengdu, Sichuan, P. R. China

**Keywords:** *Gentaina urnula*, chloroplast genome, phylogenetic analysis

## Abstract

*Gentiana urnula*, is an alpine medicinal endemic to the Qinghai-Tibetan Plateau. In this study, the complete chloroplast DNA sequence of *G. urnula* was assembled，and its genome is 149,064 bp in length, including a large single-copy region (LSC) of 81,158 bp and a small single-copy region (SSC) of 17,034 bp, which was separated by a pair of 25,436 bp inverted repeat regions (IRs). A total of 130 genes are detected, including 86 protein-coding genes, 37 tRNA genes, and 7 rRNA genes. The complete chloroplast genomes of *G. urnula* will help to study the genetic diversity and phylogenetic analysis of Gentianales.

Endemic to the Qinghai–Tibetan Plateau (QTP), *Gentaina urnula* is a medicinal herb, which is distributed in the alpine meadow ecosystems at 3900–5700 m a.s.l. It is regarded as a Tibetan folk medicine for the treatment of clearing away heat and detoxifying (Zong et al. 2015). In the present study, we reported the complete chloroplast genome sequence of *G. urnula* based on the next-generation sequencing method and revealed its phylogenetic relationship with seven Gentianaceae species of which complete chloroplast genomes are available.

We collected its fresh leaves from a single individual from Lasa city (altitude 5300 m, N 30.244°, E 99.658°) and dried them with silica gels. Its voucher specimen was stored in the Sichuan University Herbarium. The complete chloroplast genomes of *G. urnula* was assembled and analyzed. Firstly, we isolated the total genomic DNA from fresh leaves using a modified hexadecyltrimethylammonium bromide (CTAB) method (Doyle and Doyle 1987) and sequenced it based on the Illumina pair-end technology. Secondly, we used the program NOVO-Plasty to assemble the filtered reads (Dierckxsens et al. 2017), and kept chloroplast-related reads by mapping reads to *Swertia verticillifolia* as the reference using Burrows-Wheeler Alignment (BWA) tool (Li and Durbin 2009) and SAMtools (Li et al. 2009). Finally, Annotations of chloroplast genome were conducted by the program Plann (Huang and Cronk 2015), and annotations were corrected manually with the Geneious (Kearse et al. 2012). The annotated complete chloroplast DNA of *G. urnula* has been deposited into GenBank with the accession number of MK602170.

The size of the complete chloroplast genome of *G. urnula* is 149,064 bp. It is a circular DNA and contains two copies of inverted repeats (IRs) (IRa and IRb, 25,436 bp) separating the large single-copy region (LSC) of 81,158 bp and the small single-copy region (SSC) of 17,034 bp. In addition, a total of 130 genes were annotated including 86 protein-coding genes, 37 tRNA genes, and 7 rRNA genes. Most genes occur as a single copy, however, 3 rRNA genes (i.e. 4.5S, 5S and 16S rRNA), 5 protein-coding genes (i.e. *ndhB*, *rpl23*, *rps12*, *ycf15*, and *ycf2*), and 7 tRNA genes (i.e. *trnA-UGC*, *trnI-CAU*, *trnI-GAU*, *trnL-CAA*, *trnN-GUU*, *trnR-ACG*, and *trnV-GAC*) occur in duplicated copies. The GC-content of the complete chloroplast genome is estimated to be 37.8%.

Furthermore, we selected eight species from Gentianaceae to ascertain the *G. urnula* phylogenetic position within Gentianale using *S. verticillifolia* as an outgroup. All the sequences were first aligned by MAFFT (Katoh and Standley 2013). Then the maximum-likelihood (ML) tree was reconstructed using RAxML (Stamatakis 2014) with 1000 bootstrap replicates. The result of the phylogenetic analysis indicates that *G. urnula* is close to *G. stipitata* (Figure 1).

**Figure 1.**
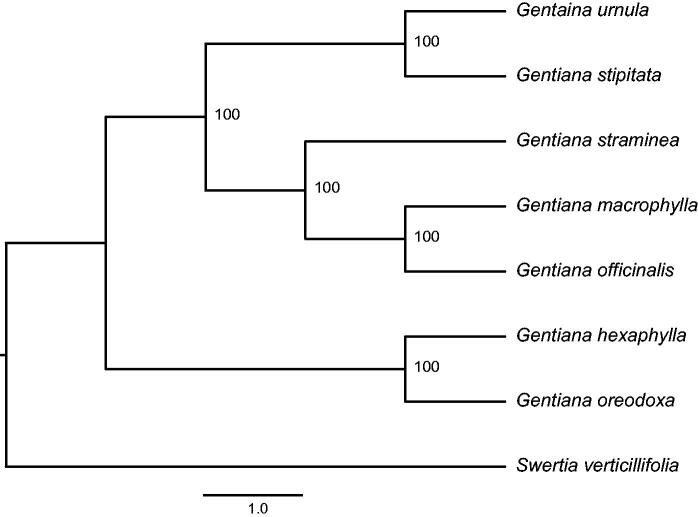
Phylogenetic relationship of the *G. urnula* chloroplast genome with seven previously reported complete chloroplast genomes. Bootstrap percentages are indicated for each branch. GenBank accession numbers: *Gentiana hexaphylla* (NC_037980.1), *Gentiana macrophylla* (NC_035719.1), *Gentiana officinalis* (NC_039574.1), *Gentiana oreodoxa* (NC_037982.1), *Gentiana stipitate* (NC_037984.1), *Gentiana straminea* (NC_027441.1), and *Swertia verticillifolia* (MF795137.1).
